# Un cas de rétinopathie pigmentaire avancée

**DOI:** 10.11604/pamj.2015.22.214.5394

**Published:** 2015-11-10

**Authors:** Fatima Zohra El Meriague, Rajae Daoudi

**Affiliations:** 1 Université Mohammed V Souissi, Service d'Ophtalmologie A de l'Hôpital des Spécialités, Centre Hospitalier Universitaire, Rabat, Maroc

**Keywords:** Rétinite pigmentaire, nyctalopie, cônes, batônnets, retinitis pigmentosa, nyctalopia, cones, rod

## Image en medicine

Il s'agit d'une patiente de 18 ans, qui présente depuis 2 ans une baisse progressive de l'acuité visuelle et une nyctalopie au niveau des 2 yeux. A l'examen, l'acuité visuelle au niveau de l’œil droit est à 1/10 et au niveau de l’œil gauche à 2/10. Le tonus est à 12 mmhg. L'examen du segment antérieur est normal. L'examen du fond d’œil montre une rétinopathie pigmentaire très étendue. La rétinite pigmentaire constitue un groupe de troubles oculaires héréditaires rares des photorécepteurs ou de l’épithélium pigmentaire de la rétine entrainant une perte progressive profonde de la vision ou une cécité. Les manifestations cliniques sont très variables: une nyctalopie, une réduction du champ visuel, des troubles de la vision des couleurs et des photopsies. L’évolution est très lente sur plusieurs dizaines d'années. Quand la maladie est présente à la naissance, elle peut être confondue avec l'amaurose congénitale de Leber et la dystrophie des cônes. Il n'existe pas, à l'heure actuelle, de traitement permettant de guérir de la rétinite pigmentaire. Quelques précautions peuvent ralentir la progression de la maladie. Le port de verres protecteurs et filtrants adaptés, protégeant de la luminosité et des rayons ultraviolets est recommandé. Leur but est surtout de diminuer la sensation d’éblouissement. Un apport en vitamine A et E pourrait ralentir l'altération des cellules impliquées, les cônes et les bâtonnets. Cet effet bénéfique reste encore très discuté par la communauté médicale et scientifique.

**Figure 1:**
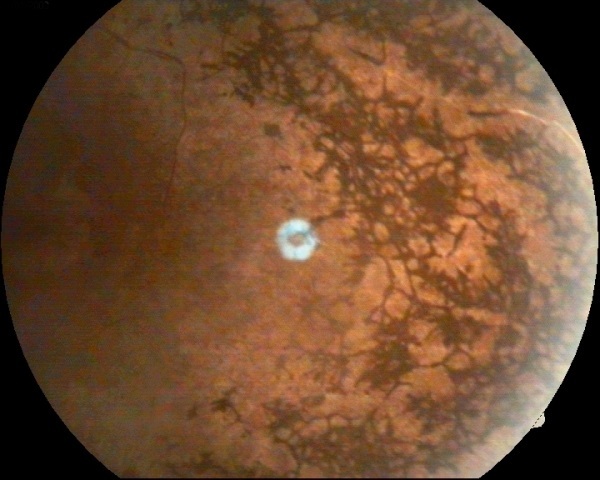
Fond d’œil montrant une rétinite pigmentaire avancée

